# A clinical observational study of effectiveness of a solid coupling medium in extracorporeal shock wave lithotripsy

**DOI:** 10.1186/s12894-022-01001-y

**Published:** 2022-04-12

**Authors:** Hao-Han Chang, Yu-Chih Lin, Ching-Chia Li, Wen-Jeng Wu, Wen-Chin Liou, Yusen Eason Lin, Kuo-Kuang Huang, Wei-Chuan Chen

**Affiliations:** 1 Department of Urology, Kaohsiung Medical University Hospital, Kaohsiung, Taiwan; 2 Graduate Institute of Clinical Medicine, College of Medicine, Kaohsiung Medical University, Kaohsiung, Taiwan; 3 Division of General Internal Medicine, Kaohsiung Medical University Hospital, Kaohsiung, Taiwan; 4 Department of Surgery, St. Joseph Hospital, Kaohsiung, Taiwan; 5 Graduate Institute of Human Resource and Knowledge Management, National Kaohsiung Normal University, Kaohsiung, Taiwan; 6 CleanWave Medical Co., LTD, Kaohsiung, Taiwan; 7 Department of Pharmacy and Master Program, Tajen University, No. 20, Weixin Rd., Yanpu Township, Pingtung County 90741 Taiwan; 8 Division of Urology, Department of Urology, Kaohsiung Veterans General Hospital, No. 386, Dazhong 1st Rd., Zuoying Dist., Kaohsiung City, 813414 Taiwan

**Keywords:** Coupling, Air pockets, Coupling medium, Extracorporeal SW lithotripter

## Abstract

This study aimed to investigate clinical effectiveness of stone disintegration by using isolation coupling pad (“icPad”) as coupling medium to reduce trapped air pockets during extracorporeal shock wave lithotripsy (ESWL). Patients underwent ESWL between Oct. 2017 and May 2018 were enrolled in this clinical observational study. An electromagnetic lithotripter (Dornier MedTech Europe GmbH Co., Germany) was used in this study. Patients were divided into icPad group P1, P2 and semi-gel group C by different coupling medium. The energy level and total number of shock wave (SW) for group P1 and C was set at level 2 and 3000 and group P2 at level 3 and 2500. The successful stone disintegration rate (SSDR) was determined to evaluate the treatment outcome. All patients were evaluated by KUB film and ultrasonography after 90 days. Complications during ESWL were recorded. A total of 300 patients satisfied the inclusion criteria. There were no significant differences in characteristics of patients and stone among three groups. The corresponding SSDRs for patients in group P1, P2 and C was 73.0%, 73.2% and 55.3%, respectively. The SSDR in group P1 was statistically higher than Group C. Comparing to semi-liquid gel, coupling medium using by icPad could achieve better treatment outcome of stone disintegration in ESWL.

## Introduction

The revolution of extracorporeal shock wave lithotripsy (ESWL) provided an insight into the role of stone treatment in the current and future [1, 2]. ESWL had kept its role as a single noninvasive treatment in stone management. Yet, there is still room for improvement in treatment outcome [1, 6]. The evolved works included design of shock wave (SW) generator and focal zone, technical procedure of ramping and lowered pulse rate, target localization and adequate coupling in the past three decades [3–5].

Modality of SW transmission shifting from water bath to water-cushion has made this procedure more convenient and comfortable for patients during ESWL procedure. However, the effectiveness were not comparable to the original one [7, 8]. The reason was inadequate coupling because air pockets trapped during smearing semi-liquid gel could impair the acoustic energy transmission of SW and then significantly decreased effectiveness of stone disintegration [9]. For instance, air pockets covering 1.5–19% of coupling area would reduce amplitude reduction of 20% in SW and even 2% air coverage could decrease stone disintegration rate by 20–40% [10]. Adequate coupling became the major concern to achieve successful outcome in ESWL [11]. Resolution of coupling became a critical step to prevent transmission of SW from acoustic energy loss [12, 13].

Regarding coupling with less entrapped air pockets during the procedure, several methods were introduced. First, a large volume of gel dispensing directly onto the head of lithotripter could diminish the amount of entrapped air pockets [10]. This technique using applying a bolus of gel to the treatment head might remove the air bubbles in an in vitro study [14]. Second, the Optical Coupling Control (OCC) system, which equipped with an inline camera for air pockets observation, could help operator to repeat the coupling procedure and achieve less air-pockets coupling [15–17]. Third, coupling a solid gel disc would cause less air bubbles as an alternative option [18, 19]. Further, a proprietary isolation-coupling pad (“icPad”) had demonstrated its superior efficacy of stone disintegration by markedly reducing trapped air pockets during coupling in a phantom study [20]. Given the advantages of icPad’s efficacy in stone disintegration, the aim of this study was to further investigate the clinical effectiveness for stone disintegration in patients undergoing ESWL.

## Materials and methods

**Fig. 1:**
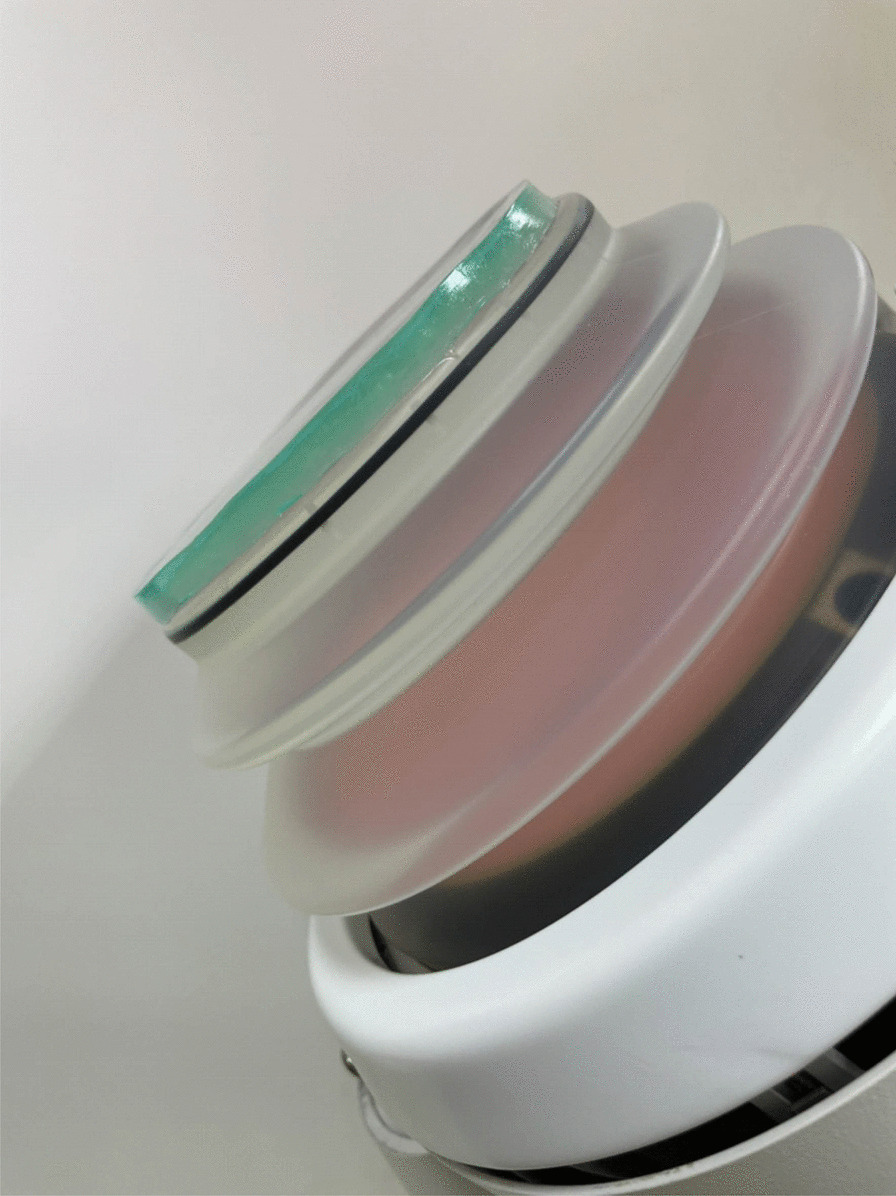
A proprietary IcPad (blue color) fit tightly on the treatment head

**Fig. 2:**
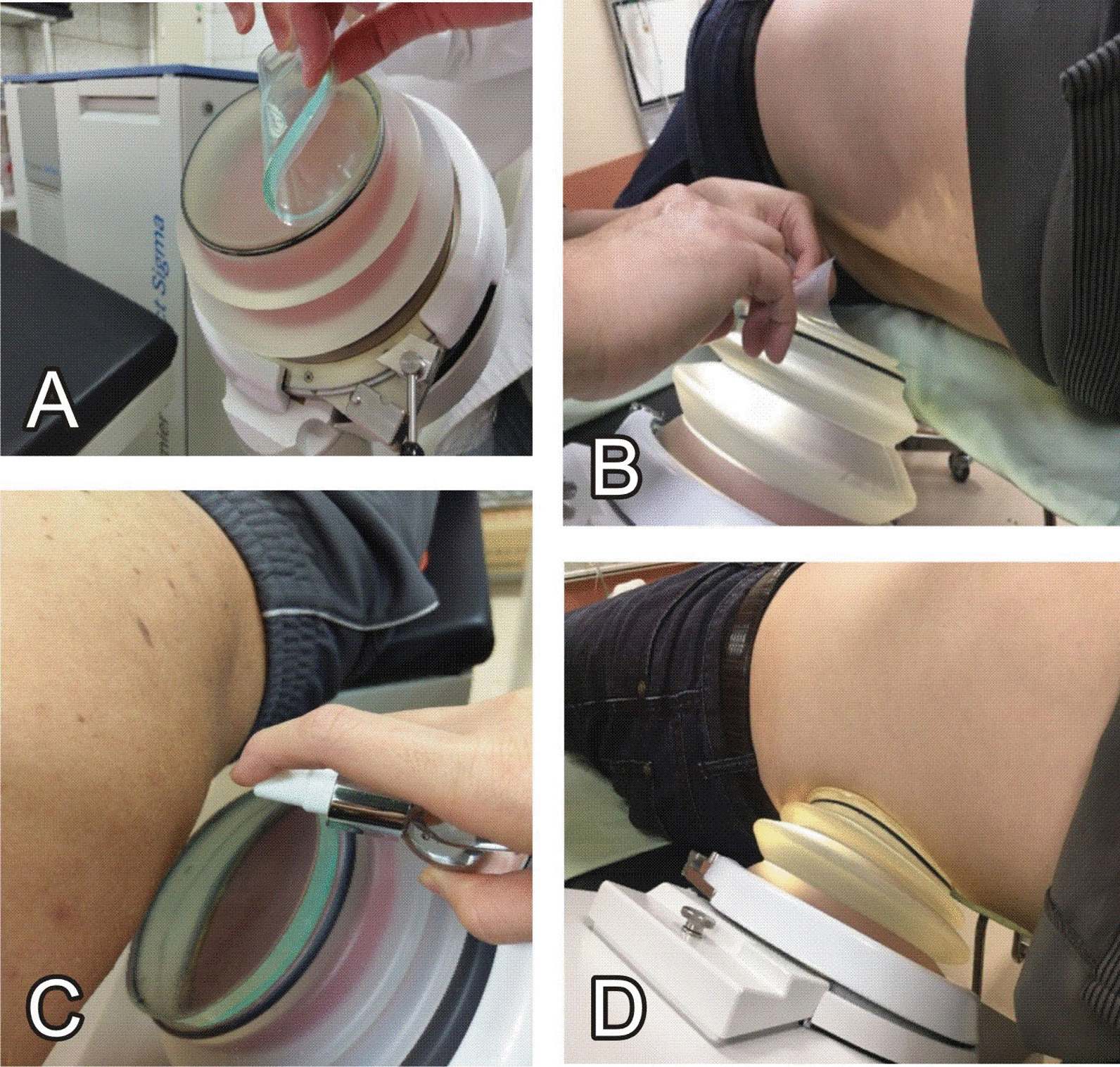
The procedure of applying icPad. **A** Paste the icPad gently to the head of lithotripter, **B** Remove the cover on the body side of icPad following probe side pasted. **C** Spray lubricant to surface of body side before moving bellow to the body. **D** Complete coupling before starting ESWL. The whole procedure can be completed in about 2 min

During Oct. 2017 to May. 2018, patients with ureteral or renal stones were eligible for this study by KUB, ultrasonography or non-enhanced computed tomography. An electromagnetic lithotripter (Dornier MedTech Europe GmbH Co., Germany) was used. The coupling medium were icPads (Diameter = 150 mm, Thickness = 8 mm) consisting of chemical-gel, mainly polyacrylamide (Fig. 1) and standard semi-liquid gel (Sonogel®) widely used in clinical practice. The procedure of applying icPad was demonstrated in Fig. 2 (Fig. 2).

A total of 300 patients were enrolled in this study. 100 patients were treated in the Group P1, 97 patients in the Group P2, and 103 in Group C. The patients were divided into three groups (Group P1, P2 and C) according to different coupling medium (icPad or semi-liquid gel) and lithotripsy settings (energy lever and total number of SW) (Table 1). All treatments were performed by attending urologists and assisted by an experienced nurse. Before the treatment, patient’s medical history, physical examination, urine analysis and radiologic investigation were performed. Characteristics patients and treated stones were recorded. Stone free (SF) was defined as complete absence of stone fragments and clinical insignificant residual disintegration (CIRD) was defined as stone burden less than 4 mm on KUB examination after ESWL. Successful stone disintegration rate (SSDR) of each group was calculated as the patients of SF and CIRD/total patients of each group and was used to evaluate the treatment effectiveness. All patients were evaluated by both KUB film within 90 days after ESWL to measure operative outcome and stone burden. Ultrasonography was used to detect hydronephrosis or major renal trauma was highly suspected. Complications were recorded during or after ESWL.

**Table 1:** Treatment parameters of ESWL

	Group C N = 103	Group P1 N = 100	Group P2 N = 97
Coupling medium	Semi-liquid gel	icPad	icPad
Total SW numbers/session	3000	3000	2500
Rate of pulse of hock (number/min)	90	90	90
Energy level	3	3	3
Fluoroscopy time (s)	347.1 ± 159.2	370.9 ± 158.1	311.3 ± 114.4
Treatment time (min)	39.36 ± 3.48	39.55 ± 3.79	41.53 ± 3.13

Chi-square test, one-way ANOVA were used for categorical and numerical variables. Statistical significance was set at *p* < 0.05. IBM SPSS 26.0 (IBM Corp., Armonk, NY) was used for all statistical analyses.

## Results

There were no significant differences in characteristics of patients and treated stones among three groups (Table 2). The chi-square test revealed that treatment outcomes (SSDR) were significantly different between group P1 and group C (73.0% vs. 55.3%, *p* = 0.009), but not significantly different between group P1 and P2 (*p* = 0.975) (Table 3). No major organ complications were noted in each group.

**Table 2:** Patients’ and stones’ characteristics

Parameters	Group C	Group P1	Group P2	F value	*P* value
Number of patients	103	100	97		
Age (years)	52.5 ± 12.4	51.4 ± 11.1	50.4 ± 10.9	0.845	0.431
Stone size (mm)	8.14 ± 3.46	7.94 ± 2.84	8.14 ± 3.12	0.134	0.875
Stone side (No./%)					
Left	59 (57.3%)	60 (60.0%)	56 (57.7%)		
Right	44 (42.7%)	40 (40.0%)	41 (42.3%)		
Stone location(No./%)					
Kidney	47 (45.6%)	50 (50.0%)	47 (48.5%)		
Upper ureter	25 (24.3%)	28 (28.0%)	24 (24.7%)		
Middle ureter	4 (3.9%)	0 (0.0%)	3 (3.1%)		
Lower ureter	27 (26.2%)	22 (22.0%)	23 (23.7%)		

Comparison performed by ANOVA test

**Table 3:** Successful stone disintegration rate (SSDR) of each treatment the groups

	≤ 4 mm		> 4 mm		Total	X^2^	*p*-value
	n	%	n	%			
Group C	57	55.3	46	44.7	103	6.872	.009
Group P1	73	73.0	27	27.0	100		

	≤ 4 mm		> 4 mm		Total	X^2^	*P*
	n	%	n	%			
Group P1	73	73.0	27	27.0	100	.001	.975
Group P2	71	73.2	26	26.8	97		

Comparison performed by Chi-square test

## Discussion

In 1983, the first lithotripter became available to treat urinary stones extracorporeally. It was discovered that there was very low energy dissipation when SW energy traveled through water [21]. The coupling system in this lithotripter provided excellent SW energy transmission, yet, the patient was required to be submerged in a water tank [22]. The procedure was inconvenient and made patients uncomfortable. Later, coupling in dry head lithotripsy the was invented and could make the patient positioned on a table without wetting the whole body. However, it did not provide the similar effectiveness due to decreased energy of transmitted SW [7, 23]. The effectiveness of different coupling design revealed that SW transmission through water could provide better outcome of stone disintegration [7, 8, 23]. The reason is the presence of “trapped air pockets” in coupling could reduce the acoustic transmission of energy by reflecting SW. Therefore, removing the air pockets in coupling gel were pivotal for effectiveness of stone disintegration [16, 24, 25].

Both techniques of applying a bolus of gel and assistance of OCC were introduced to decrease the presence of the entrapped air pockets during coupling [10, 26]. Better SFR as well as less total numbers were demonstrated in the patients undoing ESWL by the assistance of OCC [19]. However, smearing larger gel was still an operator-dependent technique and the higher cost using OCC might hinder its wide use by healthcare providers. In our previous phantom study, icPads had demonstrated better coupling and higher efficacy of stone disintegration than semi-liquid gel (92.3% vs. 45.5%) [20]. The area of trapped air pockets observed in coupling using icPad was only 0.38%, which was significantly lower than that of semi-liquid gel (2.55%). Even after sliding patient on the treatment table during ESWL procedure, air pockets only increased as little as 0.54%. Given the superior efficacy of icPad in stone disintegration in our phantom study [20], this study aimed to further investigate the clinical effectiveness for stone disintegration by lowering the total number of SW to 2500 in group P2. The SSDR of group P1 or P2 (73.0% vs. 73.2%) was higher than group C (55.3%). After running Chi-square test for comparison SSDR (stone burden ≤ 4 mm) among 3 groups, the results demonstrated that treatment outcome using icPad was better than semi-liquid and lowered total number of SW could achieve the similar outcome in icPad groups. It also indicated that the work life of lithotripter generator might be prolonged by applying less number of SW.

This is the first clinical observational study to investigate the effectiveness of the newly designed icPad. However, the study was conducted at single center and further studies are needed to validate our results. Another limitation is that this study is actually a convenience sampling. Thus, opportunity to participate is not equal for all qualified individuals.

## Conclusions

To our knowledge, this is the first clinical observational study to investigate the effectiveness of coupling medium using a solid gel pad during ESWL procedure. The advantages of icPad demonstrated that better patient outcome of stone disintegration could be achieved at lower number of SW and energy level. Our encouraging results suggested that the icPad as a coupling medium might be a cost-effective solution for future lithotripters.

## Data Availability

All data generated or analysed during this study are included in this published article.
