# Correction to: A clinical observational study of effectiveness of a solid coupling medium in extracorporeal shock wave lithotripsy

**DOI:** 10.1186/s12894-022-01124-2

**Published:** 2022-11-07

**Authors:** Hao‑Han Chang, Yu‑Chih Lin, Ching‑Chia Li, Wen‑Jeng Wu, Wen‑Chin Liou, Yusen Eason Lin, Kuo‑Kuang Huang, Wei‑Chuan Chen

**Affiliations:** 1 Department of Urology, Kaohsiung Medical University Hospital, Kaohsiung, Taiwan; 2 Graduate Institute of Clinical Medicine, College of Medicine, Kaohsiung Medical University, Kaohsiung, Taiwan; 3 Division of General Internal Medicine, Kaohsiung Medical University Hospital, Kaohsiung, Taiwan; 4 Department of Surgery, St. Joseph Hospital, Kaohsiung, Taiwan; 5 Graduate Institute of Human Resource and Knowledge Management, National Kaohsiung Normal University, Kaohsiung, Taiwan; 6 CleanWave Medical Co. Ltd., Kaohsiung, Taiwan; 7 Department of Pharmacy and Master Program, Tajen University, No. 20, Weixin Rd., Yanpu Township, 90741 Pingtung County, Taiwan; 8 Division of Urology, Department of Urology, Kaohsiung Veterans General Hospital, No. 386, Dazhong 1st Rd., 813414 Zuoying Kaohsiung, Taiwan

**Correction to: Chang et al. BMC Urology (2022) 22:56**

10.1186/s12894-022-01001-y

Following publication of the original article [1], the authors identified an error in affiliation information of corresponding author Dr. Wei-Chuan Chen.

The affiliation information has been updated above and the original article [1] has been corrected.

